# Degradation Mechanisms in Quantum-Dot Light-Emitting Diodes: A Perspective on Nondestructive Analysis

**DOI:** 10.3390/ijms262110465

**Published:** 2025-10-28

**Authors:** Hyunho Lee

**Affiliations:** Department of Electronic Engineering, Kwangwoon University, Seoul 01897, Republic of Korea; hyunho@kw.ac.kr

**Keywords:** quantum-dot light-emitting diodes, operational lifetime, degradation mechanisms, nondestructive analysis, exciton dynamics

## Abstract

Quantum-dot light-emitting diodes (QLEDs) have emerged as promising candidates for next-generation display technologies owing to their high color purity and external quantum efficiency. Despite rapid advancements in device performance, operational stability and long-term reliability remain critical challenges, particularly for cadmium-free and blue-emitting QLEDs. This review provides a comprehensive overview of the degradation mechanisms of QLEDs, emphasizing the relationship between environmental factors, such as moisture, oxygen, and thermal stress, and excitonic factors, including charge-injection imbalance, Auger recombination, and interface deterioration. We further highlight the role of nondestructive characterization techniques, including impedance spectroscopy, Fourier transform infrared spectroscopy, transient photoluminescence, transient electroluminescence, transient absorption, and electroabsorption spectroscopy, in probing real-time charge dynamics and material degradation. By integrating the insights from these operando analyses, this review offers a detailed perspective on the origins of device degradation and provides guidance for rational design strategies aimed at enhancing the operational stability and commercialization potential of QLEDs.

## 1. Introduction

### 1.1. Quantum-Dot Physics

**Figure 1:**
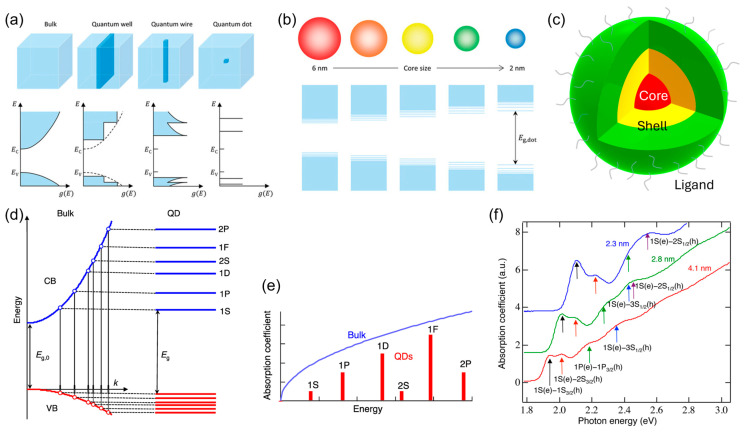
(**a**) Quantum-confinement effect on dimensions and (**b**) quantum-dot size-dependent energy-bandgap variation (Reproduced with permission) [5]. Copyright 2024, Wiley-VCH. (**c**) Structure of quantum dot. (**d**) Electronic states in a bulk semiconductor and quantum dot with their energy bandgaps. (**e**) Absorption characteristics of a bulk semiconductor and quantum dot. (**f**) Absorption coefficient with respect to photon energy. Arrows indicate energy transitions within discrete quantum-dot levels (Reproduced with permission) [4]. Copyright 2016, American Chemical Society.

Quantum dots (QDs) are semiconductor materials that exhibit a quantum-confinement effect and an electro-optical phenomenon that arises when their physical dimensions (a few to tens of nanometers) are reduced below the exciton Bohr radius. Figure 1a,b show the quantum-confinement effect of the QDs with their size distribution. Size-dependent bandgap tuning in QDs enables a straightforward modulation of their emission-spectrum peaks. QDs can be engineered to cover the three primary colors (red, green, and blue) required for display color reproduction as well as a broader spectral range from ultraviolet to infrared. This versatility enables their application in diverse optoelectronic fields such as displays, photovoltaics, and photodetectors.

Following the initial report on the quantum-confinement effect by Rossetti et al. in 1983, colloidal synthesis was realized within the subsequent decade and continuous structural refinements culminated in the widely adopted core/shell architecture [1,2,3]. Figure 1c depicts the general structural configuration of QDs comprising the core, shell, and surface ligands. The core governs the absorption and emission processes of the QDs and is a key target for size-dependent bandgap engineering. The shell physically passivates the core and energetically confines excitons, as exemplified by type-I QDs. QDs are categorized as type-I, type-II, and quasi-type-II structures depending on the energy-level alignment between the core and shell [4]. Ligands provide shell passivation and facilitate the solution processability of QDs.

The idealized electronic-state model of colloidal QDs is shown in Figure 1d. The quantized states (such as 1S, 1P, and 1D) represent discrete energy levels. These levels correspond to the states allowed for charge carriers and exhibit a discrete arrangement that is markedly different from the continuous E–k diagrams of conventional bulk semiconductors. This gives rise to the intrinsic absorption spectrum of QDs, based on the quantum-confinement effect, which exhibits characteristic absorption peaks, as shown in Figure 1e,f. When integrating colloidal QDs into electronic devices, electron and hole injection as well as lossless confinement must be carefully considered. Satisfying these conditions enables the maximization of the optoelectronic efficiency of QD-based devices. From this perspective, we discuss the recent progress in electroluminescent QD light-emitting diodes (QLEDs) employing the colloidal QDs described above.

### 1.2. QLEDs

**Figure 2:**
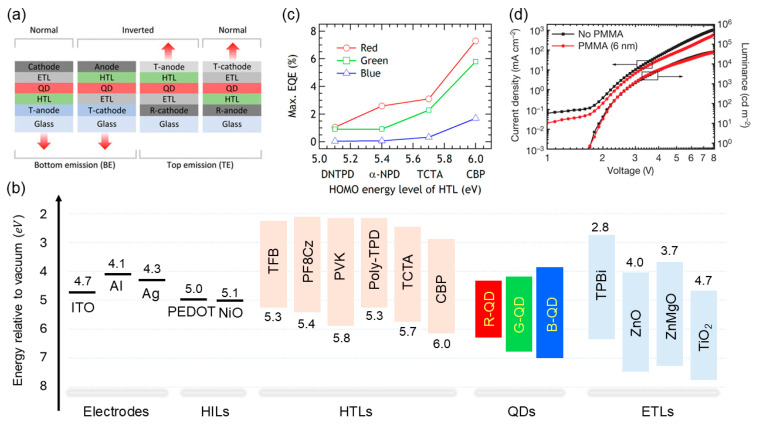
(**a**) Device structure of QLEDs (Reproduced with permission) [5]. Copyright 2024, Wiley-VCH. (**b**) Energy levels of charge-transport layers, electrodes, and QDs. The full names of undefined abbreviations are listed. ITO: indium tin oxide, PF8Cz: poly[(9,9-dioctylfluorenyl-2,7-diyl)-alt-(9-(2-ethylhexyl)-carbazole-3,6-diyl)], PVK: poly(9-vinylcarbazole), Poly-TPD: poly(4-butyltriphenylamine), TCTA: tris(4-carbazoyl-9-ylphenyl)amine, CBP: 4,4′-Bis(N-carbazolyl)-1,1′-biphenyl, TPBi: 2,2′,2″-(1,3,5-Benzinetriyl)-tris(1-phenyl-1-H-benzimidazole). (**c**) external quantum efficiency (EQE) of QLEDs with respect to the HOMO (highest occupied molecular orbit) level of the HTLs (hole transporting layer) (Reproduced with permission) [14]. Copyright 2012, American Chemical Society. (**d**) J-V-L characteristic of QLEDs upon the insertion of thin poly(methyl methacrylate) layer (Reproduced with permission) [15]. Copyright 2014, Nature Publishing Group.

Since the first report on an electroluminescent colloidal QLED in 1994, device engineering has advanced toward improving the efficient injection of charge carriers into the QD emissive layer [5,6,7,8,9,10]. Electroluminescent QLEDs exhibit structural variations designed for specific purposes, as illustrated in Figure 2a. Centered on the QD emissive layer, hole-injection and -transport layers are positioned between the anode and QDs, whereas electron-injection and -transport layers are located between the cathode and QDs. Depending on the direction in which the emitted light exits the device, QLEDs are classified as bottom- or top-emission structures. The architecture is inherently flexible and varies based on research objectives. Efficient device operation relies on the appropriate energy-level alignment among the constituent layers. Figure 2b shows the energy-level distribution of the QDs in the visible-emission range, along with the commonly used electrodes and charge-transport layers in QLEDs. Efficient charge injection requires the offset between the conduction-band minimum of the QDs and electron-transport layer, as well as between the valence-band maximum of the QDs and hole-transport layer to be minimized. Such an alignment allows efficient electron and hole injection into the QDs under an applied electric field with minimal energy loss. The intrinsically deep valence band of QDs has made improved hole injection a key characteristic of the reported studies [11,12,13]. Accordingly, addressing the insufficient hole-injection rate relative to the more facile electron injection has been a major challenge. In 2012, Kwak et al. reported the introduction of an inverted architecture and hole-transport layers based on small molecules with deep HOMO levels, which significantly increased device efficiency (Figure 2c) [14]. The high EQE, based on the HOMO distribution of the HTL, requires the improvement of the hole-injection rate to achieve a balance with electron injection. Dai et al. reported that inserting a thin insulating polymer between the QD layer and electron-transport layer suppresses electron injection, enabling an EQE of 20% and underscoring the charge-injection balance as a key factor in device engineering (Figure 2d) [15]. Current EQEs have surpassed the theoretical limits, with reported values of 30.9%, 28.7%, and 21.9% for red, green, and blue emissions, respectively [16,17]. Moreover, employing a top-emission architecture along with a light-scattering layer can boost the EQE to 44.5% [18]. Furthermore, the introduction of tandem structures results in an EQE of 51.2% [19]. QLEDs have been commercialized as light sources because of their highly efficient emission characteristics.

One of the critical challenges for QLED commercialization lies in the environmental impact of QD materials. In the visible emission range, Cd-based QDs exhibit exceptionally high luminous efficiency due to strong exciton confinement within the core [20]. However, Cd-based QDs face commercialization difficulties because cadmium is restricted under the restricted hazardous substances (RoHS) directive. Therefore, the introduction of environmentally benign QDs is essential [21,22]. InP-core-based red and green QLEDs have been developed with remarkably high efficiency and operational lifetime [23,24,25]. Nevertheless, due to the intrinsic energy band limitations of InP, synthesizing defect-controlled QDs capable of emitting blue light remains highly challenging. As an alternative, ZnTeSe ternary QD cores have been developed, leading the progress in blue-emissive light sources [26,27].

## 2. Stability and Degradation Mechanisms in QLEDs

The critical bottleneck in the commercialization of QD electroluminescent displays is the limited operational stability of the devices. Although QD-based technologies continue to demonstrate remarkable progress in terms of color purity and efficiency, their device lifetimes do not meet the requirements of display applications. For organic light-emitting diodes (OLEDs), a T50 lifetime of 56,000 h at an initial luminance of 1000 cd/m^2^ has been reported for blue emission, which represents the lowest operational stability among the three primary colors (red, green, and blue) [10,28]. The wavelength-dependent differences in operational stability (particularly the limited stability of blue emission) have resulted in display artifacts such as burn-in and image retention. Various strategies have been employed in display design to mitigate these issues, including enlarging the area of the blue subpixels, adopting tandem device architectures, and implementing compensation algorithms, which inevitably lead to unnecessary resource consumption. The differences in operational stability among wavelengths are even more pronounced in QLEDs. Although red and green emissions from QLEDs have been reported to exhibit highly stable lifetimes (e.g., T95 lifetimes exceeding 17,000 h at an initial luminance of 1000 cd/m^2^), these QDs typically employ cadmium in their cores (which falls under RoHS regulations), which limits their commercialization [12,29]. Furthermore, Cd-free blue-emitting QLEDs exhibit lifetimes that require urgent improvement (T50 = 442 h at an initial luminance of 650 cd/m^2^) [26]. Improving operational stability requires an understanding of the underlying physics of the factors affecting the device, which can be broadly classified into external influences and excitonic factors arising from device operation. Compared with OLEDs, QLEDs still suffer from interfacial instability and charge imbalance that accelerate nonradiative recombination and material degradation during operation. In this section, we provide a progress report on the operational lifetime of QLEDs and review the related degradation mechanisms.

### 2.1. Progress of Reported Operating Lifetime

Table 1 presents a progress report on the operational stability of QLEDs as a function of emission wavelength. The table summarizes the types of QD core materials, initial luminances, and operational lifetimes.

**Table 1:** Progress report on the operational stability of QLEDs.

Emission Color	Year	Core Material	Initial Luminance (cd/m^2^)	Operational Lifetime (h)	Reference
Red	2014	CdSe	100	104,000 (T50)	[15]
	2015	CdSe	100	300,000 (T50)	[30]
	2019	CdSe	100	1,600,000 (T50)	[31]
	2019	ZnCdSe	100	1,800,000 (T50)	[16]
	2021	CdSe	100	125,000,000 (T50)	[32]
	2022	InP	100	32,000 (T95)	[33]
	2022	InP	100	110,000 (T50)	[34]
	2023	CdSe	1000 * 100	6360 (T95) * 318,755 (T95)	[35]
	2024	CdZnSe	100	1,100,000 (T95)	[29]
	2025	CdZnSe	1000 * 100	61,180 (T95) * 3,066,263 (T95)	[36]
Green	2019	CdSe	100	1,700,000 (T50)	[31]
	2020	ZnCdSe	100	1,655,000 (T50)	[37]
	2022	CdSe	100	2,570,000 (T50)	[17]
	2022	InP	100	20,044 (T50)	[38]
	2024	ZnCdSe	100	10,000,000 (T50)	[39]
	2024	InP	100	508,000 (T50)	[24]
	2024	CdSe	100	18,000,000 (T50)	[12]
	2025	InP	100	293,052 (T50)	[40]
Blue	2020	ZnTeSe	100	15,850 (T50)	[26]
	2022	CdZnSe	100	24,000 (T50)	[17]
	2023	ZnCdSe	100	80,377 (T50)	[41]
	2024	CdZnS	100	41,022 (T50)	[42]
	2025	ZnSeTeS	100	30,000 (T50)	[43]
	2025	CdZnSe	1000 * 100	54 (T95) * 2706 (T95)	[44]

* Calculated with acceleration factor (*n* = 1.7) with ${L_{0}}^{n}\times T=constant$, where $L_{0}$ represents initial luminance.

### 2.2. Degradation Mechanisms

QLED degradation is driven by various factors. In particular, exposure to moisture and oxygen can induce intrinsic material degradation, leading to shifts in energy levels or diminished charge-transport properties. Furthermore, nonradiative recombination can occur at interfaces within the device during charge transport and recombination processes. Such nonradiative pathways give rise to undesirable side effects, including Joule heating. Excessive charge carriers can be driven by an electric field to induce degradation of the device components. Auger recombination, in which injected charge carriers within the QDs excite each other to induce nonradiative recombination, represents a major loss mechanism in QLEDs and is known to accelerate device degradation under high-luminance (high-current) operation. These degradation factors are not independent; however, they are interrelated and synergistically accelerate device degradation. In this section, we classify them as environmental and electrically driven factors to examine the mechanisms underlying device degradation.

The understanding of degradation mechanisms provides direct guidelines for device engineering. For instance, charge injection imbalance, which accelerates exciton-induced degradation, can be mitigated by introducing charge transport layers with selective carrier blocking or by tuning the energy-level alignment to achieve balanced electron–hole injection. Similarly, ion migration and interface reactions can be suppressed by employing more stable interlayers or robust encapsulation structures. These insights bridge the understanding of degradation physics with practical engineering strategies, guiding the rational design of device stacks and materials for improved operational stability.

#### 2.2.1. Environmental Factors

**Figure 3:**
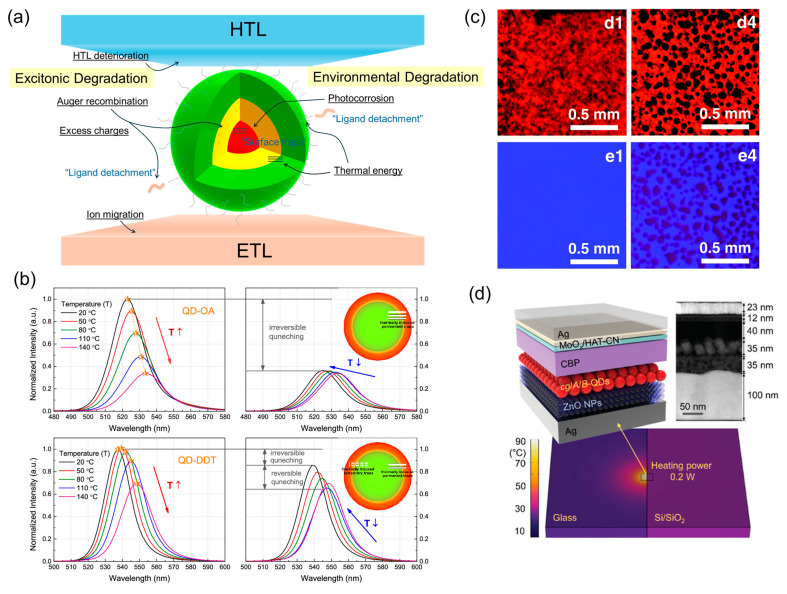
(**a**) Degradation mechanisms of QLEDs based on external and excitonic factors. (**b**) Thermal stability of the QDs upon ligand addition (Reproduced with permission) [48]. Copyright 2019, American Chemical Society. (**c**) EL images of QLEDs (d1 and d4) and optical microscopy images of poly(9,9-dioctylfluorene-alt-N-(4-sec-butylphenyl)-diphenylamine) (TFB) films (e1 and e4) after device operation for 1.5 h (Reproduced with permission) [49]. Copyright 2022, Royal Society of Chemistry. (**d**) Temperature difference of operating QLEDs upon substrates (Reproduced with permission) [32]. Copyright 2022, Wiley-VCH.

Exposure to oxygen and moisture results in adsorption onto QD surfaces, deteriorating their optoelectronic performance. Figure 3a shows representative mechanisms for the instability of the QDs. Photoexcited QDs undergo photocorrosion upon such exposure, generating surface-trap states and causing photoluminescence (PL) quenching and spectral changes [45,46]. Additionally, thermal energy significantly reduces the PL intensity of the QDs [47]. Figure 3b illustrates the influence of ligands on the thermal stability of the QDs. Notably, surface ligands on QDs can detach irreversibly under thermal stress, acting as exciton-quenching sites, thereby reducing the PL lifetime. The use of strongly bound thiol-based ligands has been suggested as a strategy to mitigate thermal degradation [48]. The degradation of the QD core accelerates the operational lifetime decay. Organic materials within the device also represent a vulnerable component, as these soft materials are particularly susceptible to external factors such as moisture, oxygen, and heat. The commonly used organic HTL TFB undergoes morphological changes when exposed to moisture (Figure 3c). The emission area of the device decreases with the formation of dark spots, closely correlating with changes in the TFB morphology. This phenomenon is attributed to the electrochemical reduction in TFB and associated decrease in hole mobility [49]. The tris(4-carbazoyl-9-ylphenyl)amine HTL undergoes physical degradation during device operation because of Joule heating arising from nonradiative recombination in the emissive layer. This leads to the formation of pinholes, which act as charge-leakage pathways and exciton-quenching sites, thereby accelerating device degradation [50]. The thermal energy generated by nonradiative recombination, such as Joule heating, is exacerbated under high-luminance operation. As shown in Figure 3d, effective heat dissipation is critical for maintaining device efficiency and operational stability at high current densities. By replacing glass with sapphire, Lee et al. effectively dissipated heat and markedly enhanced device stability [32,48,51].

As mentioned earlier, external factors such as oxygen, moisture, and thermal energy accelerate device degradation. Encapsulation physically blocks the penetration of these external elements into the device, based on low WVTR (water vapor transmission rate) and OTR (oxygen transmission rate) criteria [52]. Typically, a glass or flexible lid is used in combination with an epoxy resin. For next-generation display processes based on low-temperature and flexible fabrication, thin-film encapsulation (TFE) has been extensively studied.

In display device encapsulation, process considerations such as low thermal stress and contamination from impurities are important. OLEDs employ multi-stacked organic/inorganic thin films as TFE, maintaining a low WVTR of approximately 10^−6^ [53,54]. In QLEDs, Si-based multi-layer TFE structures have been introduced, demonstrating significant lifetime improvements [55]. The water repellent spray coating based Al_2_O_3_ capsulated within self-assembly monolayers has improved operational lifetime of the devices significantly [56]. Furthermore, reports on inorganic multi-stacked TFE structures incorporating blue-range cut-off functionality suggest the potential for TFEs with anti-reflective properties that induce selective optical reflection in displays [57].

An anomalous increase in efficiency and operational lifetime in QLEDs has been reported as a positive aging effect. Several studies have investigated the origin of this phenomenon. The acidic resin used for device encapsulation has been identified as a major source of the aging effect. Joe et al. further revealed the underlying mechanism, focusing on the behavior of ZnMgO [58]. Over the aging period, favorable agents in the resin infiltrate through the constituent layers of the device, effectively passivating oxygen vacancies in ZnMgO and thereby significantly reducing exciton quenching at these vacancy trap sites. Furthermore, Jin et al. recently reported that the formation of water molecules is responsible for the positive aging effect in the devices [59]. Pre-formation of water molecules in ZnMgO led to a significant enhancement of the operational lifetime from 8 h ($T_{95}$ at the initial luminance of 9827 cd/m^2^) to 100 h ($T_{95}$ at the initial luminance of 14,653 cd/m^2^).

Although encapsulation effectively prevents external moisture and oxygen ingress, it cannot fully mitigate thermally induced degradation during operation, which must be addressed through device engineering. Via shell engineering, nonradiative pathways can be minimized by incorporating materials such as continuous-gradient shell QDs that suppress Auger recombination, mitigating heat generation during device operation [20,32,60,61]. Consequently, degradation mechanisms driven by environmental factors do not act independently but are coupled with charge dynamics during device operation, collectively accelerating device deterioration. Therefore, in the following subsection, we examine the degradation mechanisms associated with excitonic factors during device operation.

#### 2.2.2. Excitonic Factors

The deep valence-band maximum of visible-light emissive QDs, especially Cd-based systems, intrinsically limits hole injection. This mismatch results in a charge-injection imbalance under a bias, which typically manifests as the accumulation of excess electrons within the QD layer. Owing to their high energy, excess electrons under an applied field may either accelerate Auger recombination (accompanied by heat generation) or induce physical degradation at the HTL interface [5,10,62].

**Figure 4:**
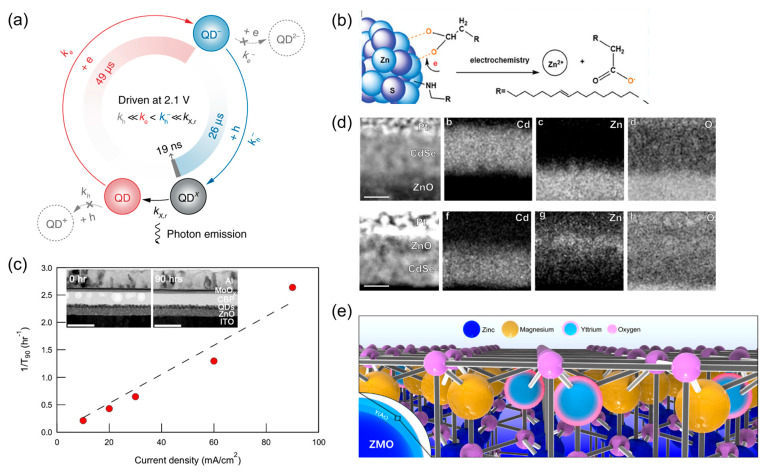
(**a**) Emission dynamics of an individual QD (Reproduced from [63], CC BY 4.0). (**b**) Ligand-detachment mechanism with device degradation (Reproduced with permission) [67]. Copyright 2024, Wiley-VCH. (**c**) Rate of degradation as a function of the applied current density (the inset shows the cross-sectional transmission electron microscopy (TEM) images of the devices as prepared and after degradation) (Reproduced with permission) [62]. Copyright 2018, American Chemical Society. (**d**) Cross-sectional high-angle dark field micrographs of QDs on ZnO and ZnO on QD films. The corresponding atomic percentage maps of Cd, Zn, and O (Reproduced with permission) [71]. Copyright 2022, American Chemical Society. (**e**) Schematic of an yttrium-passivated ZnMgO crystal structure (Reproduced from [73], CC BY 4.0.).

Many studies attribute QLED degradation to unbalanced charge injection, where excess carriers drive nonemissive recombination and physical damage. Accordingly, clarifying the detailed operating mechanics is critical for understanding the origin of device degradation. Deng et al. constructed a single-nanocrystal spectroscopy system that revealed the charging and discharging processes of QDs during their operation. The photon-emission time was orders of magnitude shorter than that of full exciton dynamics. Their study demonstrated that QDs are predominantly negatively charged via electron injection (positively charged QDs are rarely observed) and that these negatively charged QDs are emitted by subsequently accepting holes [63,64]. Figure 4a shows the emission sequence of the QDs. Kim et al. introduced a novel double emission layer (EML) device architecture to determine the location of the dominant exciton population during operation. They further performed precise calculations of the carrier mobility within the QD layer under a bias, providing representative parameters for the charge-transport behavior [65]. Ryu et al. built on the double EML structure and employed transient analysis to elucidate the emission sequence at the initial stage of QLED operation. They demonstrated that, upon voltage application, electrons reaching the HTL interface exert Coulombic attraction to the accumulated holes at the interface, thereby facilitating hole injection into the QDs [66]. Understanding the charge-injection and exciton-recombination dynamics provides critical insights into the mechanisms of device degradation.

The presence of excess electrons, dictated by the energy-level distribution of the device, can induce the detachment of surface ligands from the QDs during operation. The resulting vacant sites act as exciton-quenching centers, serving as direct evidence of device degradation [66]. Gao et al. reported that in blue-emitting QLEDs, in which electron accumulation is particularly severe, excess electrons in the QD layer detach from the oleic-acid ligands. As shown in Figure 4b, the interaction between the electrons and ligands induces ligand ionization, ultimately leading to detachment [67].

Direct evidence indicates that the overflow of excess electrons into the HTL leads to interfacial degradation [50,66]. Chang et al. reported that electrons can break molecular chemical bonds in the HTL, inducing the cross-linking of the molecules, as shown in Figure 4c [62]. Furthermore, electrons can directly compromise the hole injection layer (HIL) interfaces, including poly(2,3-dihydrothieno-1,4-dioxin)-poly(styrenesulfonate) (PEDOT:PSS), highlighting the necessity for robust HIL properties for long-term device stability [68,69].

The nanoscale dimensions of the QDs contribute to their degradation owing to the potential formation of structural leakage paths (Figure 4d). For example, when ZnO nanoparticles (approximately 5 nm) are deposited on QDs (approximately 10 nm), they can penetrate the QDs and make direct contact with the HTL. This may create shunt paths, leading to a leakage current and Joule heating, thereby accelerating device degradation [70,71].

To improve charge-injection balance, ZnMgO (with a lower mobility and higher conduction band minimum than ZnO) has been widely used in QLEDs to limit electron injection [72]. ZnMgO preserves the ZnO wurtzite structure, with Mg partially substituting Zn in the vacant lattice sites. Complete substitution is limited by the ionic-radius mismatch, and Mg ions may occasionally detach from the nanoparticles. Choi et al. reported that detached Mg ions provide the key evidence of QLED device degradation [73]. Mg detachment from ZnMgO enhances oxygen vacancies, increases exciton-quenching sites, and can further lead to Mg migration into adjacent layers during operation. Surface passivation using large-radius metal ions was proposed to alleviate these effects. Figure 4e shows the lattice-vacancy substitution of ZnMgO nanoparticles by yttrium ions.

Transparent electrode degradation serves as further evidence of device deterioration, with metal-ion migration into the QD layer observed during operation. High electric fields exacerbate this effect, causing morphological changes in the devices [74,75]. Real-time assessments of exciton-driven degradation during device operation remain rare. The next section focuses on these measurements and nondestructive techniques for evaluating device deterioration.

## 3. Characterization Methods for Nondestructive Analysis

Characterization of devices before and after degradation is crucial for understanding the underlying mechanisms. Cross-sectional scanning electron microscopy (SEM) and TEM, commonly employed to observe morphological changes, enable the direct visualization of damaged regions. Degradation is generally accompanied by physical and chemical changes, making the analysis of chemical-bond energies particularly informative. Consequently, compositional techniques such as depth-profiling X-ray photoelectron spectroscopy, time-of-flight secondary ion mass spectrometry, and Auger electron spectroscopy have been widely employed. However, analyzing degradation ex situ can lead to artifacts because ambient exposure during measurement may contaminate sensitive samples. This is particularly critical for moisture- or oxygen-sensitive devices; thus, the interval between the degradation and analysis must be carefully managed. In QLEDs, degraded devices exposed to moisture or oxygen can deteriorate much faster than newer devices, potentially leading to the overestimation of degradation. Therefore, direct physicochemical analyses must be complemented with nondestructive in situ characterization methods.

**Figure 5:**
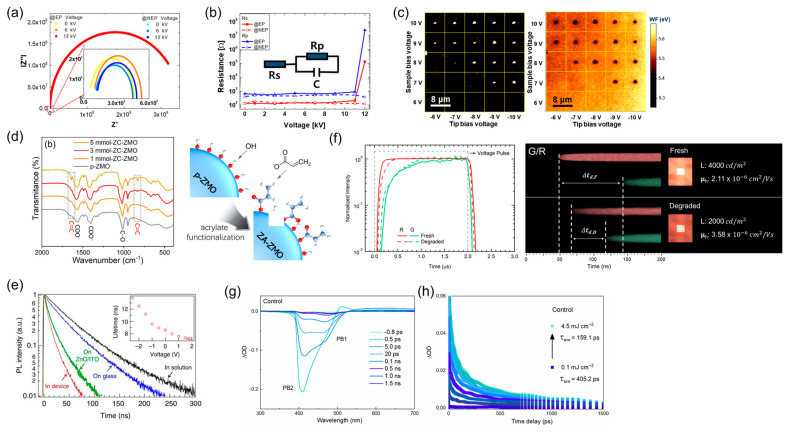
(**a**) Cole–Cole plot and (**b**) corresponding resistance changes of devices with electrostatic discharge (Reproduced from [80], CC BY 4.0.). (**c**) Topographic and work function images of QLEDs with a stable contact force and various voltage biases (Reproduced with permission) [75]. Copyright 2023, IOP Publishing. (**d**) FTIR spectra of ZnMgO nanoparticles with different ligands (Reproduced with permission) [94]. Copyright 2022, American Chemical Society. (**e**) Time-resolved photoluminescence (TRPL) spectra of QDs on different substrates. The inset shows the TRPL spectra of the devices at different voltage biases (Reproduced from [97], CC BY 4.0.). (**f**) Time-resolved electroluminescence (TREL) of QLEDs and hole mobility in devices upon operational degradation (Reproduced with permission) [66]. Copyright 2025, American Chemical Society. (**g**) Transient absorption (TA) spectra at selected timescales for QDs, and (**h**) bleach dynamics of QDs at different excitation densities (Reproduced from [25], CC BY 4.0.).

Impedance spectroscopy is a practical technique that provides an in situ probe for materials, heterojunctions, and devices under alternating voltages [76]. Capacitance–frequency measurements effectively probe charge accumulation and recombination in QLEDs, with accumulation dominating at high frequencies and recombination dominating at low frequencies. Capacitance–voltage analysis provided further information on the charge buildup and device depletion regions [77,78,79]. Figure 5a,b show the impedance analysis of QLED degradation based on electrostatic discharge. Lee et al. reported on the mechanisms of QLED failure under transient electrostatic stress. Degradation involves migration of the Al cathode, which alters the internal charge distribution. Notably, the expansion of the Nyquist plot based on the equivalent circuit provides a direct indication of the device degradation [80]. The negative capacitance observed in C–V measurements provides important information on the charge dynamics in organic electronic devices and QLEDs [81,82]. Huang et al. reported that shifts in negative capacitance in QLEDs are directly attributed to degradation caused by hole traps [83]. While impedance spectroscopy provides valuable insights into charge transport and recombination dynamics, the extracted parameters are often sensitive to device geometry, frequency range, and fitting models. As a result, inconsistent interpretations have been reported across studies, suggesting that a standardized protocol for measurement and data analysis is still needed.

Conventional atomic-force microscopy (AFM) is a morphology-based and potentially destructive technique owing to mechanical probing; however, derivative methods such as conductive AFM and kelvin probe force microscopy enable the in situ investigation of device degradation via tip-based measurements [84,85,86]. Using local electric fields, Cha et al. induced local field-driven degradation in QLEDs by observing morphological damage accompanied by work-function changes. In particular, In-ion migration from an indium tin oxide electrode in degraded areas led to a downward shift in the work function (Figure 5c) [75]. Operando scanning kelvin probe microscopy analysis along the vertical direction of the QLEDs revealed changes in the surface potential before and after degradation, providing in situ insights into the internal electric-field strength. Notably, degradation is associated with increased resistance at the electron transporting layer (ETL)–cathode interface [87]. AFM- and SKPM-based approaches enable high spatial resolution mapping of surface potential and morphology changes during device operation. However, their applicability to buried interfaces remains limited, and potential tip-induced perturbations can alter the very surface states being probed. These limitations underscore the importance of correlating AFM data with complementary optical or electrical analyses.

Fourier transform infrared (FTIR) spectroscopy probes molecular vibrational modes by analyzing the absorption of infrared light. It is widely employed as a nondestructive tool to identify chemical bonds and molecular structures within a sample [88]. FTIR efficiently probes the chemical stability and compositional changes in QLED interfacial and emissive layers, including QD-surface ligand modifications, organic-layer degradation, and alterations from ligand exchange [89,90,91,92]. The electroluminescent performance of QLEDs is strongly influenced by the properties of ZnO used as the ETL. As shown in Figure 5d, FTIR effectively reveals the surface chemical modifications of ZnO and enables the correlation of these chemical sites with charge traps or exciton-quenching centers [35,72,93,94]. Chrzanowski et al. used FTIR to monitor changes in the hydroxyl groups on ZnMgO surfaces, revealing the impact of air exposure on the EL performance during QLED fabrication. Controlled exposure to humid air enhanced the device-lifetime stability [95]. He et al. applied reflection-mode FTIR spectroscopy to quantify the acetate on ZnO surfaces as an indicator of QLED degradation. The C = O stretching vibration at 1600 cm^−1^ served as a marker, with concentrations determined via the Lambert–Beer law. The accumulation of acetate byproducts under the cathode during operation provided direct evidence of device degradation [87]. Gao et al. provided FTIR evidence for ligand detachment in QDs as a mechanism of blue-QLED degradation. Oleic-acid ligands on QDs were identified in the 1540–1413 cm^−1^ region, and changes in the corresponding FTIR signals indicated that accumulated electrons in the QDs facilitated ligand detachment [67]. Ryu et al. employed FTIR as direct evidence that excess charge carriers induce ligand detachment in QLEDs. Changes in the Zn–S stretching vibration, representing 1-dodecanethiol ligands in the 400–600 cm^−1^ region, were observed, demonstrating ligand loss from QD surfaces during device degradation [66].

Transient analyses are effective for investigating exciton dynamics on the nanosecond scale. TRPL, which is commonly employed in optoelectronic devices, directly probes emissive layer dynamics, offering clear insights into the effects of the emissive layer and adjacent interfaces on degradation [58,72,96]. Figure 5e shows the transient PL decay of QDs under several conditions. Bae et al. applied TRPL with a biased voltage ranging from the negative to the sub-turn-on range. The decreasing trend of the average PL lifetime with increasing bias voltage indicates the presence of a high Auger-recombination density within the QD layer. The suppression of Auger recombination was directly observed as a steady level of the PL lifetime with bias-voltage variation [97]. Kim et al. intentionally induced excessive charge states within the QD layer of InP QLEDs via structural modifications. When hole carriers accumulated as excess charges, the TRPL decay was significantly reduced, which was further verified using hole-only devices and the corresponding TRPL measurements. They attributed the permanent device degradation to the deterioration of QD surfaces by excess holes [98]. Ryu et al. configured a QD layer as a double-emissive color structure and performed simultaneous TRPL measurements at green- and red-emission wavelengths (520 and 630 nm, respectively) to study the device degradation. They analyzed the TRPL lifetimes at each wavelength by varying the device structures, including hole-only and electron-only devices as well as QD-containing architectures. Degradation mechanisms such as ligand detachment have been shown to be directly correlated with TRPL-lifetime decay [66].

TREL provides the detailed temporal responses of EL under a voltage-pulse bias, enabling a comprehensive analysis of charge injection and recombination within devices. In particular, for OLEDs, the EL time delay in response to the applied voltage was used to extract the minority carrier mobility as a physical parameter [99]. Furthermore, the decay of EL after the voltage pulse was turned off was used to analyze the charge accumulation [100]. TREL has broad applications in the analysis of various planar EL devices [101]. Kim et al. applied transient EL analysis to a double EML structure (red- and green-emitting QDs) to examine the EL response times under a periodic voltage bias. Temporal differences between red and green emissions allowed the determination of the lower bounds for charge mobility within the QD layer during device operation. Furthermore, the EL decay was fitted exponentially to extract the average TREL decay time, which was correlated with the presence of trap sites at the interfaces adjacent to the QDs [65]. Similarly to OLEDs, the TREL-based analysis of minority carrier mobility has been applied to QLEDs. Doe et al. examined three HTL variants by measuring the EL rise delays to determine the hole mobility. They observed a decline in hole mobility during degradation and discussed its effects on charge-injection balance and device-lifetime stability [102]. Zhao et al. employed TREL to investigate the parasitic emissions and loss mechanisms in QLEDs. TREL measurements were conducted for red, green, and blue QLEDs, and the analysis of the delay times between the QD EL and leakage EL from the HTL provided insights into the mechanism of parasitic emission, which was attributed to interface traps [103]. Figure 5f shows the TREL of the QLEDs with respect to degradation. Ryu et al. measured the TREL characteristics of InP QLEDs before and after degradation to determine the physical parameters from EL rise delays and decay times and analyzed the underlying causes of degradation. They reported an in situ reduction in hole mobility in the QD layer during operation and correlated this with direct evidence of ligand detachment. Furthermore, the rapid decrease in the TREL decay time with device aging was attributed to the deterioration at the QD/HTL interface [66].

TA effectively probes semiconductor-property changes below specific bandgaps as it records absorption-based dynamics. In particular, it enables the precise analysis of the electron density within the target layer [104]. TA enables the precise analysis of the electron density accumulated within QDs and the electric field experienced by the QDs during operation. Furthermore, it allows the tracking of the nonradiative behavior of excess electrons [105]. Figure 5g,h show the TA spectra and bleach dynamics of the QDs. Exciton bleaching in TA reflects the absorption reduction upon QD ground-state occupation and can be used to infer the presence of trap states [25,106,107]. Rakshit et al. employed TA spectroscopy to investigate femtosecond-scale electron and hole trapping associated with Auger recombination in InP QD cores [108]. Yan et al. analyzed the exciton formation times and lifetimes by examining the rise and decay of TA bleach signals. Amplitude analysis was used to estimate exciton densities. Additionally, the electric-field-induced Stark effect was employed to probe the internal electric-field distributions, presence of exciton dipole moments, and exciton annihilation mechanisms [104]. TRPL, TREL and TA measurements provide dynamic insights into exciton recombination and defect formation. Nevertheless, most measurements are conducted under idealized or bias-free conditions, which may not fully represent the actual device environment. Future operando optical setups integrating electrical bias and ambient control could bridge this gap.

Electron absorption (EA) spectroscopy was used to probe the influence of an external electric field on the optical absorption properties of a material. The applied field induces the Stark effect, which alters the absorption spectrum by modifying the electronic states. These spectral changes can be used to assess the internal electric-field distributions, space-charge accumulation, and interlayer charge transfer within the devices. Chen et al. interpreted the quadrature component of the EA spectra as a charge-accumulation indicator. The strong quadrature signals observed in the ETL were attributed to charge buildup at the QD/ETL interface during device degradation, leading to the deterioration of the junction properties [109]. Luo et al. used EA measurements to determine the flat-band voltage of the emissive layer, which coincided with the EL turn-on voltage. They further analyzed the emission mechanism at subthreshold voltages in relation to the carrier-injection dynamics [110]. Relating the carrier-injection and emission-voltage parameters to degradation analyses can yield valuable insights.

To better summarize the nondestructive tools discussed above, Table 2 compares their measurement principles, strengths, limitations, and typical applications in QLED degradation studies. Overall, these nondestructive techniques exhibit complementary capabilities in terms of temporal resolution and sensitivity: time-resolved optical methods such as TRPL and TREL enable high temporal resolution for probing fast excitonic dynamics, whereas impedance and spectroscopic techniques provide higher sensitivity to slower interfacial or chemical degradation processes.

## 4. Conclusions and Remarks

In this review, we comprehensively explore the degradation mechanisms of QLEDs with a particular focus on nondestructive analysis techniques. Although QLEDs have advanced rapidly in terms of EQE, achieving performance levels suitable for commercial display applications, their operational stability and long-term reliability remain critical challenges, particularly for cadmium-free and blue-emitting devices.

QLED degradation is a complex phenomenon involving both environmental and excitonic factors. Moisture, oxygen, and thermal energy deteriorate the optical and electrical properties of materials, whereas electrical driving conditions lead to charge imbalance, interface damage, and nonradiative recombination such as Auger processes. These degradation pathways are not independent but interlinked, synergistically accelerating device failure.

To address these issues, recent studies have actively employed nondestructive analytical methods such as impedance spectroscopy, FTIR spectroscopy, transient PL, transient EL, transient absorption spectroscopy, and electro absorption spectroscopy. These techniques provide valuable insights into real-time charge dynamics and material degradation during device operation, thereby offering powerful diagnostic tools for reliability assessment and device engineering.

We emphasize that further progress in QLED stability will require a more comprehensive understanding of the degradation mechanisms of QLEDs. In particular, establishing clear correlations between specific degradation signatures and performance decreases, as enabled by nondestructive analyses, is crucial for the rational design of next-generation QLEDs.

This review bridges degradation science with in-operando diagnostics and can serve as a useful guide for researchers aiming to unlock the full commercial potential of QLED technologies.

Looking forward, future research should aim to integrate nondestructive analyses with operando device characterization platforms capable of simultaneously probing optical, electrical, and chemical changes. Such multimodal approaches would enable a quantitative correlation between charge dynamics and chemical degradation under realistic operating conditions. In parallel, data-driven methodologies such as machine learning-assisted spectral interpretation could accelerate the identification of degradation pathways and lifetime predictors. On the device engineering side, further advances in encapsulation strategies, charge transport layer design, and interfacial passivation are essential to mitigate the coupled effects of environmental and excitonic degradation. Ultimately, a synergistic framework that connects fundamental degradation science with practical device optimization will be key to achieving reliable, cadmium-free QLED technologies for next-generation displays.

## Figures and Tables

**Table 2:** Comparison of nondestructive analysis techniques for QLED degradation studies.

Technique	Principle/Measured Quantity	Strengths	Limitations	Typical Applications in QLEDs
Impedance Spectroscopy (IS)	Frequency-dependent response of charge transport and accumulation	Provides in situ information on charge accumulation, recombination, and device degradation; sensitive to interface processes	Limited spatial resolution; interpretation requires equivalent-circuit modeling	Identifying charge-trapping sites; monitoring cathode migration and charge imbalance during degradation
Conductive AFM/Kelvin Probe Force Microscopy (KPFM)	Local current or surface potential mapping via nanoscale probe	Spatially resolved electrical/morphological correlation; can visualize local degradation and work-function shifts	Tip-induced mechanical damage possible; limited temporal resolution	Mapping degraded spots, work-function shifts at ETL/cathode interface, local field-induced degradation
Fourier-Transform Infrared Spectroscopy (FTIR)	Infrared absorption by molecular vibrational modes	Nondestructive chemical-bond identification; sensitive to surface ligand or functional-group changes	Requires IR-active species; limited depth sensitivity	Tracking QD-ligand detachment, ZnO surface hydroxyl/acetate evolution, interfacial chemical degradation
Time-Resolved Photoluminescence (TRPL)	Emission decay dynamics following pulsed excitation	Direct probe of exciton dynamics; identifies nonradiative recombination and trap formation	Requires optical transparency; may not access buried interfaces	Correlating PL lifetime with trap formation, excess charge effects, or ligand detachment during operation
Transient Electroluminescence (TREL)	Time-resolved emission under pulsed bias	In situ probe of charge injection, transport, and recombination kinetics	Requires functioning device; limited to emissive region	Evaluating charge mobility changes, interface traps, and emission delays with device aging
Transient Absorption (TA)	Photoinduced absorption change (exciton bleach dynamics)	Ultrafast temporal resolution (fs–ns); probes carrier trapping and recombination	Complex instrumentation; optical penetration depth limited	Tracking exciton bleaching, Auger recombination, and field-induced exciton dynamics
Electroabsorption (EA)	Modulation of optical absorption under electric field (Stark effect)	Directly probes internal electric fields and charge accumulation	Requires transparent electrodes; complex spectral deconvolution	Quantifying space-charge buildup, interface charge transfer, and flat-band voltage evolution

## Data Availability

No new data were created or analyzed in this study. Data sharing is not applicable to this article.
